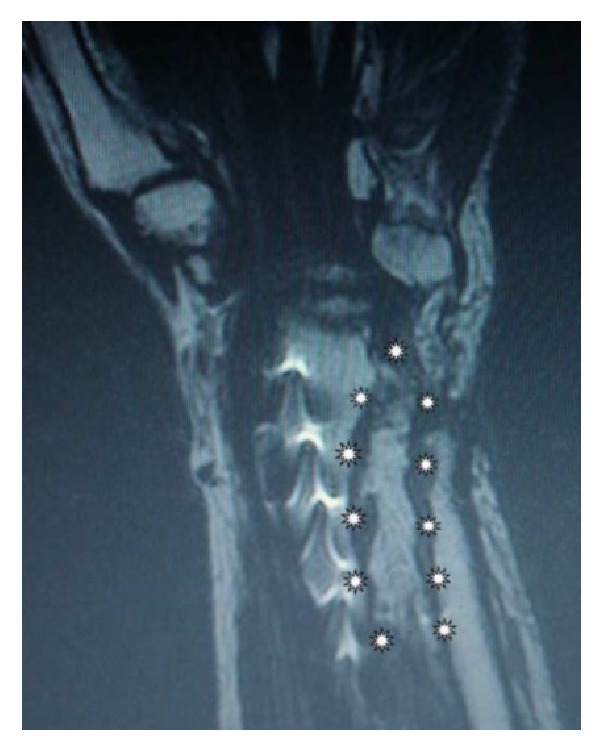# The Dorsoulnar Artery Perforator Adipofascial Flap in the Treatment of Distal Radioulnar Synostosis

**DOI:** 10.1155/2017/3271026

**Published:** 2017-07-24

**Authors:** Alessia Pagnotta, Giorgio Antonietti, Iakov Molayem

**Affiliations:** ^1^Hand and Microsurgery Unit, Jewish Hospital, Rome, Italy; ^2^Department of Orthopaedics and Traumatology, Padre Pio Hospital, Bracciano and San Paolo Hospital, Civitavecchia, Italy

## Abstract

Posttraumatic radioulnar synostosis (RUS) is a rare event following forearm fractures. Consequences are disabling for patients who suffer from functional limitation in forearm pronosupination. Distal RUS are even more rare and more difficult to treat because of high recurrence rates. The patient we describe in this paper came to our attention with a double distal RUS recurrence and a Darrach procedure already performed. We performed a radical excision of RUS and interposition with a vascularized dorsoulnar artery (DUA) adipofascial perforator flap. Four years after surgery, the patient shows the same complete range of motion in pronosupination, and MRI confirms that the flap is still in place with signs of vascularization. Simple synostosis excision has been proven ineffective in many cases. Interposition is recommended after excision, and biological material interposition seems to be more effective than foreign material. Surgeons are increasingly performing vascularized interposition, and the results are very encouraging.

## 1. Introduction

Radioulnar synostosis (RUS) in adults is a rare event, usually posttraumatic, resulting from forearm single or both bones fractures. It often leads to complete loss of forearm pronosupination, and the functional result is disabling for the patients, both in recreational activities and in everyday life.

This pathology has been classified by Vince and Miller [1] dividing it into 3 types depending on the location: type I is in the distal radioulnar area, type II in the middle nonarticular area, and type III in the proximal area. Jupiter and Ring [2] modified this classification by subclassifying type III synostosis into types A, B, and C. Type IIIA synostosis involves the bicipital tuberosity, type IIIB synostosis lies at the radial head and the proximal radioulnar joint, and type IIIC is associated with complete ankylosis of the elbow with widespread heterotopic bone. Hastings II and Graham [3] modified the classification adding 3 areas of interest and suggesting possible treatment procedures depending on the location.

There is wide consensus on the need of surgical treatment in order to restore range motion and function. Initially, procedures were focusing on synostosis excision alone [1, 4] or associated with nonvascularized biological [3, 5, 6] or artificial material interposition [1, 7]. High recurrence rates and poor results in studies with large series of patients [1, 2, 4] pushed surgeons to look for different solutions to achieve better outcomes.

Sugimoto et al. [8] and others [9–12] began to advocate the need for vascularized interposition, either pedicled muscle or adipofascial flaps, that could last through time and not cause adverse internal reactions. Distal RUS is even more rare and disabling for patients, being associated with even poorer results [1].

We describe a case that can be classified as a type I according to Vince and Miller [1] and area 5 according to Hastings II and Graham [3], with a history of double recurrence and a Darrach procedure [13] already performed, treated by synostosis resection and interposition with an adipofascial dorsoulnar artery (DUA) perforator flap.

## 2. Case Report

**Figure 1:**
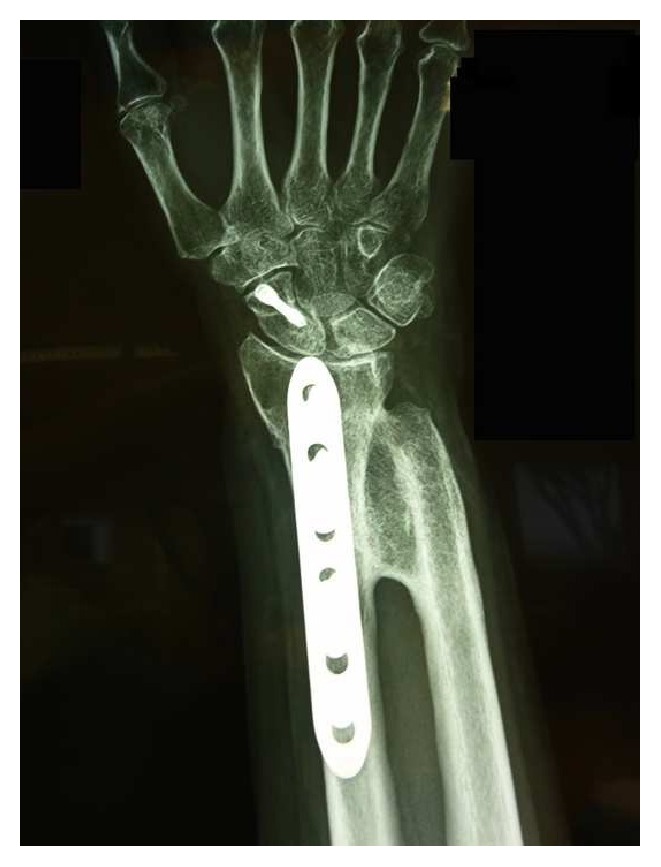


A 76-year-old woman suffered from an accidental fall, sustaining a distal forearm (both radius and ulna) and scaphoid fracture. Surgery was performed 10 days after initial trauma with scaphoid, radius, and ulna osteosynthesis. No specific NSAID were given to the patient. Assisted wrist and elbow exercises began 60 days postoperatively. She immediately noticed a complete loss of pronosupination and X-rays revealed distal RUS. Six months later, she underwent synostosis excision and Darrach procedure [13], with ulnar implants removal. Intraoperative pronosupination was complete. Rehabilitation began 1 week after surgery, but soon after she developed progressive loss of pronosupination, and 1-year postoperative X-rays revealed a recurrent synostosis (Figure 1). The patient came to our attention for the first time 1 year after the second surgery. She reported strong limitations in daily activities; she was feeling demoralized and had given up the idea of performing some of her hobbies (cooking and gardening). X-rays revealed a type I radioulnar synostosis [1]. Despite her age, the patient was completely independent, very active in everyday life, and determined to regain full forearm function.

**Figure 2:**
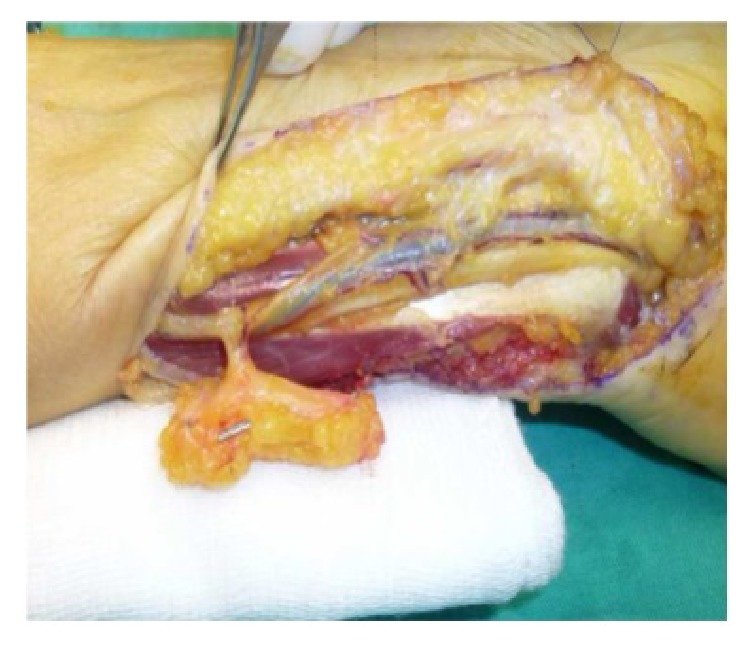


**Figure 3:**
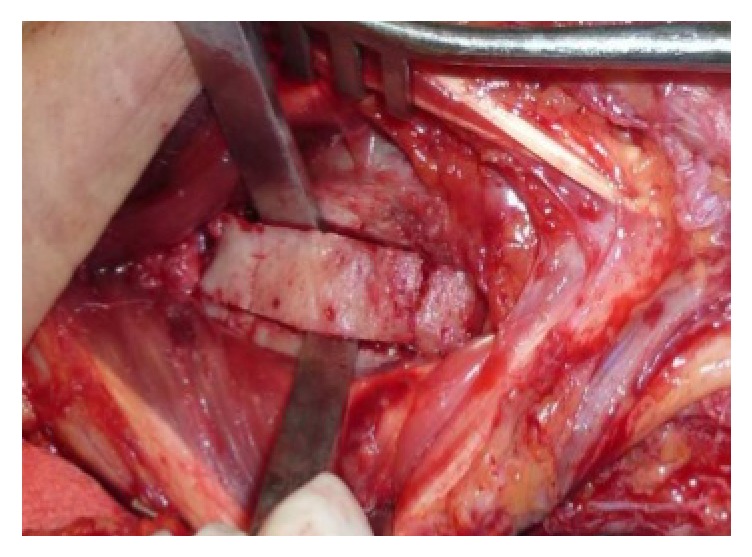


**Figure 4:**
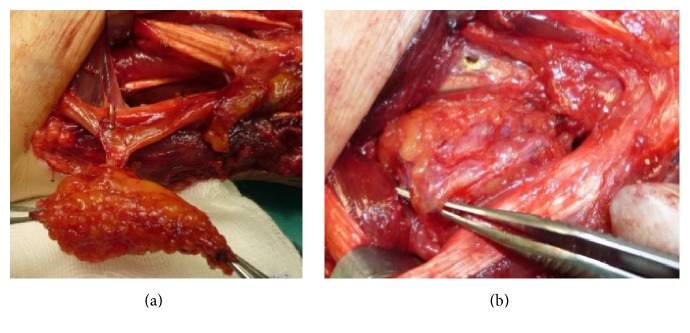


**Figure 5:**
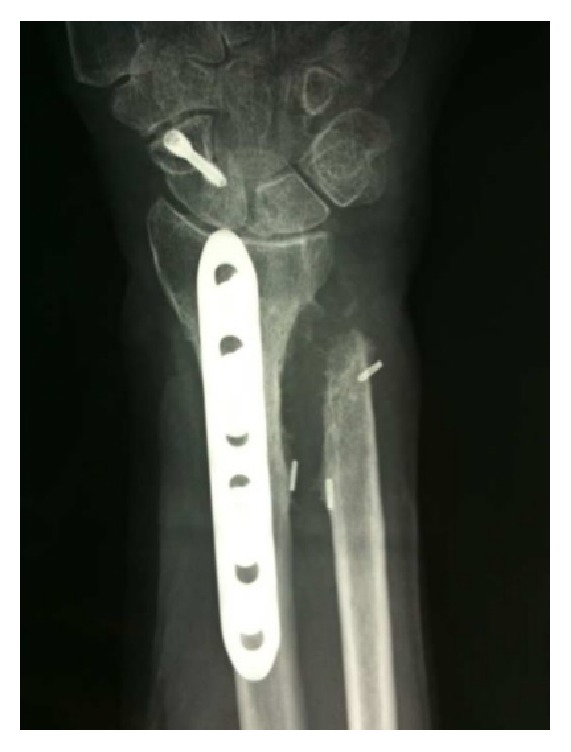


We planned a vascularized DUA perforator adipofascial flap following synostosis excision after checking the presence of the perforator vessel with Doppler ultrasound. An ulnar approach was used, and fascia was exposed with a subdermal dissection. The adipofascial flap was incised, released from both radial and ulnar side, and gently retracted from the underlying flexor carpi radialis muscle. We identified the DUA perforating vessel that was visible on the flap's distal third (Figure 2). The interosseous membrane and the synostosis were then exposed and excised (Figure 3), and the flap was turned into the distal radioulnar space (Figures 4(a) and 4(b)). Intraoperative pronosupination was complete. Active and passive range of motion exercises were started 3 days postoperatively and continued for several months. No splints, NSAID, or radiotherapy was used. Four years after surgery, the patient still shows complete pronation (90°) and supination (90°) and no synostosis recurrence (Figure 5). MRI performed 1 year after surgery confirms that the flap is still located in the interosseous space showing signs of a vascularized tissue (Figure 6).

## 3. Discussion

Radioulnar synostosis can be congenital, posttraumatic, iatrogenic, or idiopathic. Incidence of posttraumatic synostosis ranges from 1.2% to 6.6% of patients suffering from forearm fractures, but it is much higher in patients with associated brain lesions.

Our patient was affected by type I according to Vince and Miller [1] and an area 5 according to Hastings II and Graham [3] RUS with a history of double recurrence and a Darrach procedure already performed [13].

Hastings II and Graham [3] suggest performing a Sauvè-Kapanji procedure [14] for synostosis in this area, but since the patient had already undergone a Darrach [13], this would not allow us to carry out a proper procedure. Furthermore, this surgery has an inner risk of ossification of the pseudoarthrosis site [15]. Most articles in literatures report cases of proximal RUS. Even in the 3 major case series [1–3], only 4 cases of distal RUS are reported. They were all treated with excision, in 3 patients associated with a Darrach procedure [13] and in 1 with interposition of silicone rubber. In 3 occasions, recurrences were reported (75%). In Friedrich's [5] case series (13 pz), 2 distal RUS were reported. Patients were treated with synostosis resection and fascia lata interposition, autograft in the first 3 patients, and allograft in the next 10 patients to reduce the risk of donor site morbidity. No recurrence was seen. Sonderegger et al. [10] reported 6 patients (1 distal RUS) treated with pedicled adipofascial flaps (4 based on the radial artery and 2 on the posterior interosseous artery) with no synostosis recurrence. Distal RUS is more rare and occurs after distal radioulnar fractures; it is more difficult to treat [1], and it appears that patients treated with excision and no biological interposition show poorer results, although few cases are described.

Surgeons' RUS treatment choice is moving toward the use of biological material interposition. An experimental study on dogs showed conclusively the superiority of vascularized grafts over nonvascularized grafts or other foreign materials in preventing scar formation [16]. Radioulnar synostosis cases treated with vascularized adipofascial flaps are described and, although the total case series is low, results are satisfying in all patients [8–11].

We performed a vascularized adipofascial flap interposition, which we believe to be the most reliable treatment, considering the particular RUS location and the failures of previous surgeries. In particular, we decided to harvest and interpose the DUA perforator flap because it has significant advantages compared to other possible flaps, including a single skin incision, a constant vessel, and no risk of sacrificing the forearm vascularization as the flap is based on a perforating terminal branch. Finally, it is a local flap that does not need a microsurgical anastomosis but needs microsurgical experience.

Postoperative radiation therapy following resection has been described with positive results [17], but we decided not to irradiate our patient for the risk of flap damage.

RUS is a rare but highly disabling condition for patients. Treatment should be effective and prevent risk of recurrence. We believe that vascularized interposition should be routinely used and, in particular, that the DUA adipofascial perforator flap is a valuable treatment option for distal RUS.

## Figures and Tables

**Figure 6:**